# Comparison of Endoscopic Ultrasonography, Computed Tomography, and Magnetic Resonance Imaging for Pancreas Cystic Lesions

**DOI:** 10.1097/MD.0000000000001666

**Published:** 2015-10-16

**Authors:** Yoon Suk Lee, Kyu-hyun Paik, Hyung Woo Kim, Jong-Chan Lee, Jaihwan Kim, Jin-Hyeok Hwang

**Affiliations:** From the Department of Internal Medicine, Seoul National University College of Medicine, Seoul National University Bundang Hospital, Gyeonggi-do (YSL, K-HP, HWK, J-CL, JK, J-HH); and Department of Internal Medicine, Keimyung University School of Medicine, Daegu, Republic of Korea (YSL).

## Abstract

Consensus regarding which modality is optimal for the measurement of pancreas cystic lesions (PCLs) was not achieved although cyst size is important for clinical decisions. This study aimed to evaluate the properties of endoscopic ultrasonography (EUS) compared with computed tomography (CT) and magnetic resonance imaging (MRI) in measuring the size of PCL.

A total of 34 patients who underwent all 3 imaging modalities within 3 months before surgery were evaluated retrospectively. The size measured by each modality was compared with the pathologic size as a reference standard using Bland–Altman analysis and intraclass correlation coefficients (ICCs).

The mean size difference was 1.76 mm (ICC 0.86), 7.35 mm (ICC 0.95), and 8.65 mm (ICC 0.93) in EUS, CT, and MRI. EUS had the widest range of 95% limits of agreement (LOA) (−17.54 to +21.07), compared with CT (−6.21 to +20.91), and MRI (−6.82 to +24.12). The size by EUS tended to be read smaller in tail portion, while those by CT and MRI did not. When the size was more than 4 cm, the size on EUS was estimated to be smaller than on pathology (*r* = 0.492; *P* = 0.003).

Although 3 modalities showed very good reliability for the size measurement on PCL compared with corresponding pathologic size, EUS had the lowest level of agreement, while CT showed the highest level among the 3 modalities. Therefore, the size estimated by EUS has to be interpreted with caution, especially when it is located in tail and relevantly large.

## INTRODUCTION

Incidental detection of pancreas cystic lesions (PCLs) has increased since the resolution of cross-sectional imaging improved and medical checkups became widespread. The prevalence of PCL was reported to be from 2.6% by computed tomography (CT) to 13.5% by magnetic resonance imaging (MRI).^1,2^ Moreover, the risk of malignant or premalignant lesions, such as intraductal papillary mucinous neoplasms (IPMN) and mucinous cystic neoplasms, was known to be up to 47% of PCL.^3,4^ Therefore, the prediction of malignant potential for these lesions is very important and challenging. Since definitive diagnosis can be achieved only by surgical treatment, appropriate surveillance with imaging modalities is extremely important. Although several practice guidelines and studies have been reported for the management of PCL, the cystic size at diagnosis and cyst growth during follow-up has been considered with constant issues for treatment decisions.^3–8^ Furthermore, the revised Sendai guidelines of 2012 also recommended different follow-up intervals based on the cyst size, whereas it might be measured to be different according to imaging planes (coronal image or sagittal image) or imaging modality (CT, MRI, and endoscopic ultrasonography [EUS]) since the PCL was particularly irregular and oval rather than circular in shape. However, to the best of our knowledge, there is only 1 report investigating the size-measurement aspect of each imaging modality including EUS, CT, and MRI.^9^ Therefore, we undertook this study to investigate the relative properties of EUS compared with CT, and MRI in measuring the size of PCL using corresponding pathology as a reference standard.

## MATERIAL AND METHODS

### Study Design

Data were collected from patients referred for the evaluation of PCL from January 2009 to September 2013 in our tertiary teaching hospital. To be eligible for inclusion in this study, patients were required to receive all 3 imaging studies including EUS, CT, and MRI and histologic specimen which were obtained within 3 months since the size of PCL had been estimated by the imaging studies. Patients were excluded if: the imaging work-up revealed a pancreatic mass rather than a PCL; pancreatic cyst could not be verified based on our imaging studies; even though the patients underwent abdominal CT or MR, the coronal image was not reconstructed; triple-phase pancreatic protocol with thin slices was not applied to the abdominal CT; the CT or MRI was conducted after cystic fluid aspiration; and main-duct type IPMN was suspected.

EUS procedures were performed by 2 experts (JK, JHH) using mechanical or electronic radial echo-endoscopes (GF-UM2000 or GF-UE260, Olympus Medical Systems, Tokyo, Japan). Patients were lying in a left position under conscious sedation with pethidine and midazolam. The endoscopists made a resolute attempt to detect the longest dimension of the PCL through various planes. MRI examinations were performed using a 1.5-T system (Avanto; Siemens, Erlangen, Germany). CT examinations were conducted using a pancreatic protocol with coronal image-reconstruction multi-detector CT, which was required with a precontrast scan and pancreas parenchymal phase (at 40 seconds) with about 3 mm axial section thickness. All of the included patients underwent MRI and CT lying in a supine position.

This study was approved by the human subjects committee of the Seoul National University Bundang Hospital, and the requirement for informed consent was waived for this retrospective review. The study followed the ethical guidelines of the 1975 Declaration of Helsinki.

### Measurement of Pancreas Cystic Lesion

The written reports of every imaging investigation were scrutinized by the authors. For quality control, all of the original image sets of each image modality were reassessed by independent investigator who was unaware of the results of other modalities. Although EUS, CT, and MRI were conducted without a regular sequence, all of these studies were checked within 3 months. And the images were reviewed on site with the size measuring software of “ImageJ (version 1, http://imagej.nih.gov/ij/index.html),” which is an open source program developed by Wayne Rasband at the National Institutes of Health (Bethesda, MD). The maximum dimension of PCL was measured twice on the cross-section plane and coronal section plane, and the larger dimension on contrast-enhanced CT and T2-weighted MR images was selected as the size of the pancreas cyst. Otherwise, EUS was conducted through various planes with both transgastric and transduodenal approach to detect the longest dimension and the longest one was selected as EUS size. The cyst size described in the pathology report was regarded as the pathologic size of the cystic tumor, which was considered as a reference. The location of the PCL was categorized into 3 groups: head, body, and tail portion. The right aspect of the superior mesenteric vessels was defined as the head portion of the pancreas, which encompassed the uncinate process, head, and neck; the left aspect of the superior mesenteric vessels was divided in half into the body and tail.

### Statistical Analysis

The difference from size of the pathologic specimen was calculated as follows: the value of the size estimated by each image modality minus the pathologic size. The differences were described as means and standard deviation (SD). Each size from EUS/CT/MRI was compared with corresponding pathologic size using intraclass correlation coefficients (ICCs) applied with 2-way mixed model and type of consistency. ICC is usually used to assess reliability, which refers to the consistency of the repeatedly measured value for the same object.^10–12^ According to the value of ICC, the reliability between different method is categorized as follows: very good when the ICC is >0.80; moderate when the ICC is 0.60 to 0.79; and not reliable when the ICC < 0.60. Furthermore, Bland–Altman analysis was also conducted, which was designed to compare 2 methods of measurement with scatterplots.^13–16^ The mean difference (the bias) between measured values from imaging modalities and 95% limits of agreement (LOA) (±1.96 SD) was calculated. When the range of the 95% limits is smaller than the other methods, it is considered that the agreement is better than the others.^12,13^ In addition, the difference between the measured values (vertical axis) against their means (horizontal axis) was depicted with scatterplots. Subgroup analysis was performed with proven mucinous lesions including IPMN and mucinous cystic neoplasms as well. Correlation analyses with Pearson's correlation coefficient and multivariable linear regression analyses were conducted to evaluate the independent contributions of various parameters for the discrepancy in the size estimates. A parsimonious model with stepwise forward selection was applied to multivariable regression model. All the statistical analyses were performed with SPSS software (version 20.0 for Windows, IBM, Armonk, NY).

## RESULTS

### Characteristics of Pancreas Cystic Lesions

A total of 34 patients underwent all of the 3 image studies including EUS, CT, and MRI within 3 months before resection for PCL from January 2009 to June 2013. Among the patients, 18 patients (52.9%) were female and the mean age was 59.8 years. In terms of final histologic diagnosis, 15 patients (44.2%) were IPMN. The other clinical and pathologic characteristics of the cysts are shown in Table 1.

**TABLE 1:**
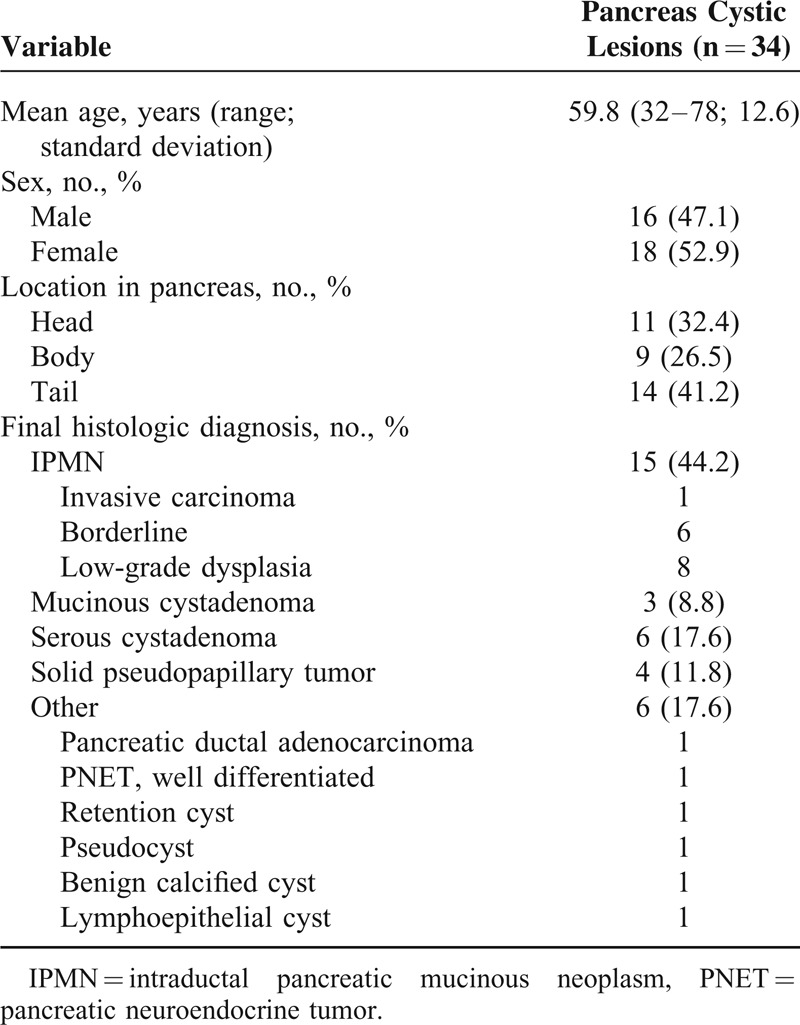
Baseline Characteristics of 34 Patients

### Discrepancy of the Size Estimates Between Each Modality and Surgical Pathologic Specimen

**FIGURE 1:**
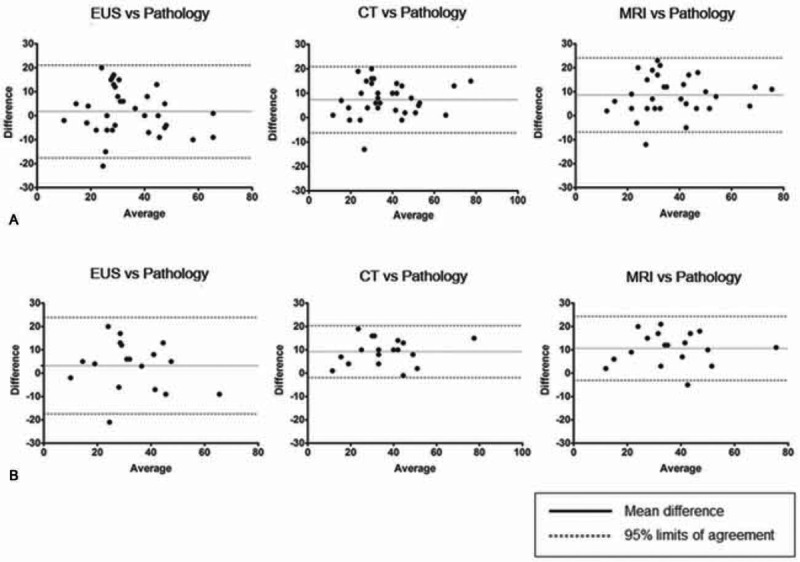
Bland–Altman analysis. (A) Total 34 patients. (B) Subgroup analysis for 18 mucinous lesions including IPMN and MCN. IPMN = intraductal pancreatic mucinous neoplasm, MCN = mucinous cystic neoplasms.

Size estimates from each imaging modality were compared to the size of the surgical pathologic specimens as a reference standard. The mean difference from the pathologic specimens (value of size measured by each image modality minus the pathologic size) was 1.76 mm in EUS, 7.35 mm in CT, and 8.65 mm in MRI. Furthermore, ICC values from 3 imaging studies for the corresponding pathologic size showed very good reliability with 0.86 in EUS, 0.95 in CT, and 0.93 in MRI (Table 2). However, Bland–Altman plots showed that EUS had the widest range of LOA among the 3 imaging tests and pancreas protocol CT had the narrowest LOA range, although all of the 3 imaging studies had good reliability with pathologic size (Fig. 1A).

**TABLE 2:**
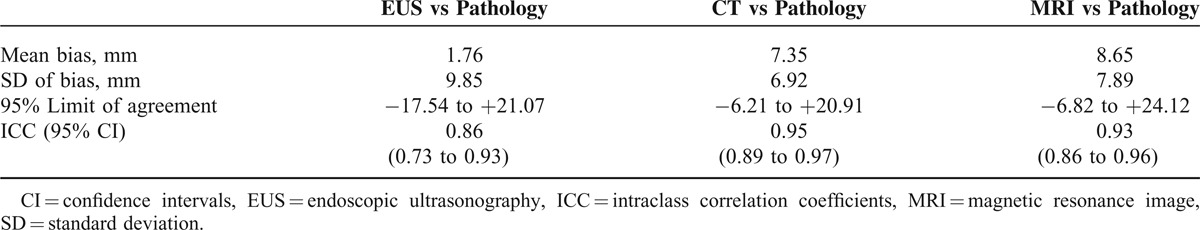
Discrepancy of the Size Estimates Between Each Modality and Surgical Pathologic Specimen

Subgroup analysis for the discrepancy in 18 mucinous lesions revealed that the mean difference from pathology was 3.22 mm in EUS, 9.22 mm in CT, and 10.61 mm in MRI. ICC values from 3 imaging studies for the corresponding pathologic size also showed very good reliability with 0.84 in EUS, 0.97 in CT, and 0.95 in MRI (Table 3). Furthermore, the widest range of LOA on EUS was continuingly demonstrated and pancreas protocol CT had the narrowest LOA range as well (Fig. 1B).

**TABLE 3:**
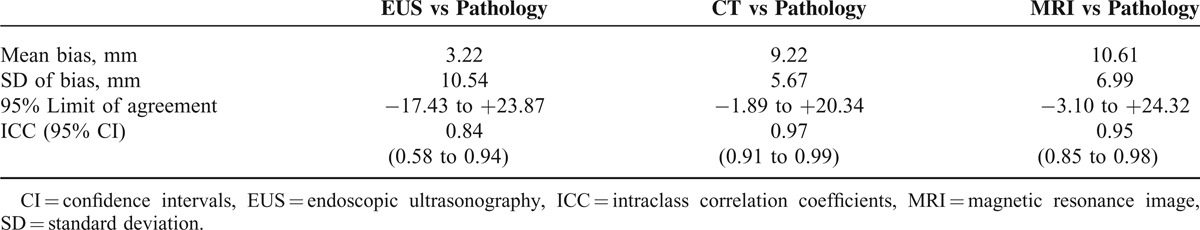
Subgroup Analysis for the Size Discrepancy in 18 Mucinous Lesions

### Contributing Factors for the Discrepancy in the Size Estimates

**FIGURE 2:**
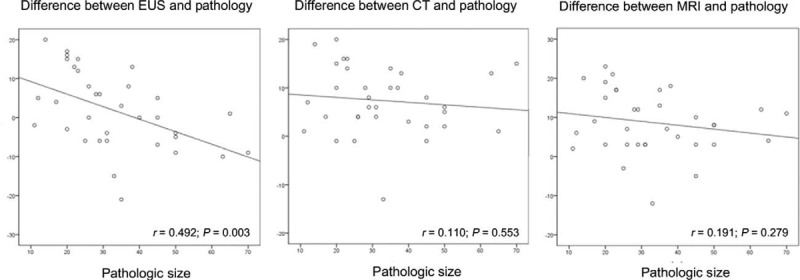
Linear regression analysis between pathologic size and the difference of size estimates from pathologic size. (A) By EUS, (B) CT, (C) MRI. CT = computed tomography, EUS = endoscopic ultrasonography, MRI = magnetic resonance image.

Linear regression analyses were performed to assess the relationship between pathologic size and the difference of size estimates by EUS/CT/MRI from pathologic size. It showed that the discrepancy decreased in EUS with the increase of cyst size. However, when the cyst size was more than about 4 cm, the size in EUS was estimated to be smaller than those in pathology using a linear regression analysis (*r* = 0.492; *P* = 0.003). In contrast, there were no significant relationships between pathologic size and the degree of discrepancy in the CT (*r* = 0.110; *P* = 0.533) and MRI (*r* = 0.191; *P* = 0.279) (Fig. 2).

**FIGURE 3:**
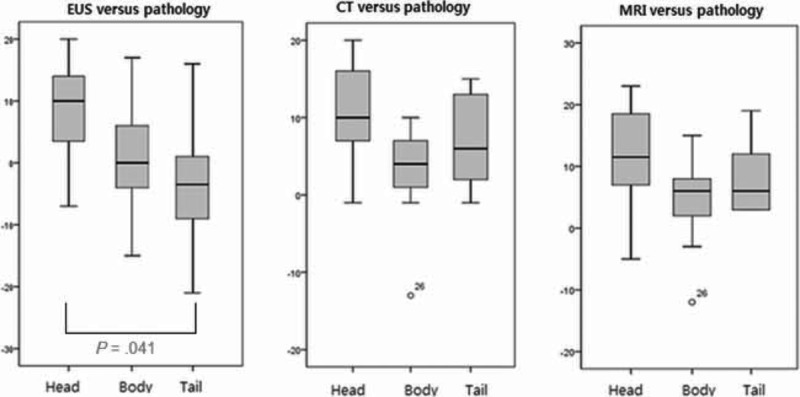
Differences between each imaging modality and pathology according to the location of pancreas cystic lesions.

Moreover, location of PCL was also analyzed for the discrepancy, which was classified into head, body, and tail portion. Although CT and MRI did not have a significant difference on the degree of discrepancy according to location, EUS had a significant difference (*P* = 0.041), which was attributed to the statistical difference between head and tail portion on post hoc analysis. The size on EUS tended to be read smaller in the tail portion, while CT and MRI did not show this tendency (Fig. 3).

## DISCUSSION

We set out to compare measurement of PCL on EUS, CT, and MRI vis-à-vis the pathologic specimens. Lesion size of ≥3 cm was regarded as worrisome feature on the 2012 revised Sendai guideline^4^ and surveillance of PCL is recommended to be carried out based on the size estimated by imaging modality. Additionally, there is a report that cyst growth of ≥2 mm/year may be a predictor for malignancy.^5^ Thus, size measurement of PCL is an important factor for clinical decision-making. However, there is no consensus statement regarding size measurement. In the present study, we found that EUS had the widest range of LOA among the 3 imaging tests, while pancreas protocol CT had the narrowest LOA range. Moreover, the size of PCL in pancreas tail by EUS tended to be underestimated and the tendency was also identified with the increase of cyst size, whereas this tendency was not identified on CT and MRI. Therefore, we suggest that cyst size estimated by EUS has to be interpreted with caution, especially when PCL is located in pancreas tail and relevantly large. To the best of our knowledge, this is the first study to demonstrate the properties of EUS compared with CT and MRI using a relatively large number of surgical specimens as a reference standard regarding the size measurement of PCL.

A variety of studies have been conducted to assess the role of each imaging modality in discriminating benign from malignant potential cysts. EUS has been regarded to have superior spatial resolution and thus have better performance when evaluating a local change and vascularity, with more fine images of inner structures of PCL,^17–20^ and differentiating small lesions.^21,22^ Furthermore, regarding the detectability of protruding lesions of IPMN, EUS was reported to be the most accurate among the 3 imaging modalities.^23^ However, there is a potential drawback to EUS, which is operator-dependent. Ahmad et al^24^ reported that agreement among experienced endosonographers for the diagnosis of PCL was little more than chance. Maimone et al^9^ also reported that EUS had a wide-range of differences when size by EUS was compared with surgical specimen. This property of EUS was also continuingly demonstrated with our present data showing the widest range of LOA among the 3 imaging modalities.

In the present study, to find out the possible factors associated with the variability, further analysis using linear regression model was conducted. Thus, it was found out that when the PCL was located in the pancreas tail or was more than about 4 cm, the size on EUS was estimated as smaller than the pathologic specimen.

There are plausible explanations for these results. First, it might be because EUS has spatial restraints for scanning the lesion from an anatomic perspective, which cause EUS to visualize a lesion at oblique angles or on different planes. On the contrary, CT and MRI are relatively free from spatial restraints for scanning the lesion and consistently use axial and coronal planes. This could also explain why the LOAs of CT and MRI were closely clustered. Second, conventional EUS is generally conducted without contrast enhancement. EUS using a gray scan may have some difficulty in delineating the margin clearly if PCL is accompanied with solid components, relatively large septum, and wall thickness because of the edge shadows. Finally, EUS is also constrained by the size and shape of PCL and transducer-frequency, which hampers visualization of the entire lesion in one imaging cut.

However, a report from Leeds et al^25^ is inconsistent with our present result. They showed that the measured sizes by CT and EUS were not significantly different from pathological size and thus, those 2 modalities are interchangeable.^25^ These inconsistent results may be attributed to methodological differences. They focused on measured values and a predictability of >3 cm by means of imaging study rather than the spread of differences from pathologic size.

These data have certain limitations. First, EUS procedures were performed with only radial echo-endoscopes although linear EUS is more popular in western countries. It might give rather homogeneity to this study by reducing the potential influence from difference between radial and linear EUS. Second, formalin fixation can cause to shrink tissue^26^ and the degree of tissue shrinkage is reported to be variable between laboratories. However, within single center the laboratory circumstance is identical for each tissue processing. Jonmarker et al^27^ reported that the average shrinkage was 4.5% in their laboratory and there was no significant difference between fixation techniques. Therefore, the pathologic size could be used as the parameter for comparative evaluation if all of those were processed within the same laboratory.

Despite the limitations, these results provide a useful insight for the properties of EUS on the size measurement of PCL. The size estimated by EUS has to be interpreted with caution, especially when PCL is located in the pancreas tail and are relevantly large. Therefore, the authors point to the need for better measurement guidelines on PCL to enhance clinical practice.

## References

[1] Laffan TA Horton KM Klein AP Prevalence of unsuspected pancreatic cysts on MDCT. *AJR Am J Roentgenol* 2008; 191:802–807. 18716113 10.2214/AJR.07.3340 PMC2692243

[2] Lee KS Sekhar A Rofsky NM Prevalence of incidental pancreatic cysts in the adult population on MR imaging. *Am J Gastroenterol* 2010; 105:2079–2084. 20354507 10.1038/ajg.2010.122

[3] Enestvedt BK Ahmad N To cease or ‘de-cyst’? The evaluation and management of pancreatic cystic lesions. *Curr Gastroenterol Rep* 2013; 15:348. 24014118 10.1007/s11894-013-0348-y

[4] Tanaka M Fernandez-del Castillo C Adsay V International consensus guidelines 2012 for the management of IPMN and MCN of the pancreas. *Pancreatology* 2012; 12:183–197. 22687371 10.1016/j.pan.2012.04.004

[5] Kang MJ Jang JY Kim SJ Cyst growth rate predicts malignancy in patients with branch duct intraductal papillary mucinous neoplasms. *Clin Gastroenterol Hepatol* 2011; 9:87–93. 20851216 10.1016/j.cgh.2010.09.008

[6] Khalid A Brugge W ACG practice guidelines for the diagnosis and management of neoplastic pancreatic cysts. *Am J Gastroenterol* 2007; 102:2339–2349. 17764489 10.1111/j.1572-0241.2007.01516.x

[7] Jacobson BC Baron TH Adler DG ASGE guideline: The role of endoscopy in the diagnosis and the management of cystic lesions and inflammatory fluid collections of the pancreas. *Gastrointest Endosc* 2005; 61:363–370. 15758904 10.1016/s0016-5107(04)02779-8

[8] Rautou PE Levy P Vullierme MP Morphologic changes in branch duct intraductal papillary mucinous neoplasms of the pancreas: a midterm follow-up study. *Clin Gastroenterol Hepatol* 2008; 6:807–814. 18304885 10.1016/j.cgh.2007.12.021

[9] Maimone S Agrawal D Pollack MJ Variability in measurements of pancreatic cyst size among EUS, CT, and magnetic resonance imaging modalities. *Gastrointest Endosc* 2010; 71:945–950. 20231021 10.1016/j.gie.2009.11.046

[10] Shrout PE Fleiss JL Intraclass correlations: uses in assessing rater reliability. *Psychol Bull* 1979; 86:420–428. 18839484 10.1037//0033-2909.86.2.420

[11] Bravo G Potvin L Estimating the reliability of continuous measures with Cronbach's alpha or the intraclass correlation coefficient: toward the integration of two traditions. *J Clin Epidemiol* 1991; 44:381–390. 2010781 10.1016/0895-4356(91)90076-l

[12] Gayat E Ahmad H Weinert L Reproducibility and inter-vendor variability of left ventricular deformation measurements by three-dimensional speckle-tracking echocardiography. *J Am Soc Echocardiogr* 2011; 24:878–885. 21645991 10.1016/j.echo.2011.04.016

[13] Myles PS Cui J Using the Bland-Altman method to measure agreement with repeated measures. *Br J Anaesth* 2007; 99:309–311. 17702826 10.1093/bja/aem214

[14] Bland JM Altman DG Statistical methods for assessing agreement between two methods of clinical measurement. *Lancet* 1986; 1:307–310. 2868172

[15] Bland JM Altman DG Agreed statistics: measurement method comparison. *Anesthesiology* 2012; 116:182–185. 22129533 10.1097/ALN.0b013e31823d7784

[16] de Vet HC Terwee CB Knol DL When to use agreement versus reliability measures. *J Clin Epidemiol* 2006; 59:1033–1039. 16980142 10.1016/j.jclinepi.2005.10.015

[17] Itoi T Sofuni A Itokawa F Current status of diagnostic endoscopic ultrasonography in the evaluation of pancreatic mass lesions. *Dig Endosc* 2011; 23 Suppl 1:17–21. 21535194 10.1111/j.1443-1661.2011.01132.x

[18] Hunt GC Faigel DO Assessment of EUS for diagnosing, staging, and determining resectability of pancreatic cancer: a review. *Gastrointest Endosc* 2002; 55:232–237. 11818928 10.1067/mge.2002.121342

[19] Song MH Lee SK Kim MH EUS in the evaluation of pancreatic cystic lesions. *Gastrointest Endosc* 2003; 57:891–896. 12776038 10.1016/s0016-5107(03)70026-1

[20] Kobayashi G Fujita N Noda Y Mode of progression of intraductal papillary-mucinous tumor of the pancreas: analysis of patients with follow-up by EUS. *J Gastroenterol* 2005; 40:744–751. 16082592 10.1007/s00535-005-1619-7

[21] Rana SS Bhasin DK Rao C Role of endoscopic ultrasound in idiopathic acute pancreatitis with negative ultrasound, computed tomography, and magnetic resonance cholangiopancreatography. *Ann Gastroenterol* 2012; 25:133–137. 24714266 PMC3959389

[22] Sadamoto Y Oda S Tanaka M A useful approach to the differential diagnosis of small polypoid lesions of the gallbladder, utilizing an endoscopic ultrasound scoring system. *Endoscopy* 2002; 34:959–965. 12471539 10.1055/s-2002-35859

[23] Baba T Yamaguchi T Ishihara T Distinguishing benign from malignant intraductal papillary mucinous tumors of the pancreas by imaging techniques. *Pancreas* 2004; 29:212–217. 15367887 10.1097/00006676-200410000-00006

[24] Ahmad NA Kochman ML Brensinger C Interobserver agreement among endosonographers for the diagnosis of neoplastic versus non-neoplastic pancreatic cystic lesions. *Gastrointest Endosc* 2003; 58:59–64. 12838222 10.1067/mge.2003.298

[25] Leeds JS Nayar MN Dawwas M Comparison of endoscopic ultrasound and computed tomography in the assessment of pancreatic cyst size using pathology as the gold standard. *Pancreatology* 2013; 13:263–266. 23719598 10.1016/j.pan.2013.02.009

[26] Schned AR Wheeler KJ Hodorowski CA Tissue-shrinkage correction factor in the calculation of prostate cancer volume. *Am J Surg Pathol* 1996; 20:1501–1506. 8944043 10.1097/00000478-199612000-00009

[27] Jonmarker S Valdman A Lindberg A Tissue shrinkage after fixation with formalin injection of prostatectomy specimens. *Virchows Arch* 2006; 449:297–301. 16909262 10.1007/s00428-006-0259-5

